# SARS-CoV-2 effect on male infertility and its possible pathophysiological mechanisms

**DOI:** 10.15190/d.2021.10

**Published:** 2021-06-30

**Authors:** Arjola Agolli, Zeynep Yukselen, Olsi Agolli, Mehrie H. Patel, Kinal Paresh Bhatt, Luis Concepcion, John Halpern, Sabaa Alvi, Rafael Abreu

**Affiliations:** ^1^Division of Clinical and Translational Research, Larkin Health System, South Miami, FL, USA; ^2^School of Public Health and Health Sciences, University of Massachusetts, Amherst, MA, USA; ^3^Larkin Community Hospital, South Miami, FL, USA

**Keywords:** Coronavirus, COVID-19, male infertility, sperm, male reproduction, fertility, ACE2, TMPRSS2.

## Abstract

First case of COVID-19 was reported in Wuhan, China in December 2019. As of now, May 2021, a total of 164,189,004 people were infected, and 3,401,990 deaths have occurred caused by SARS-CoV-2. As SARS-CoV-2 virus cell entry mainly depends on the ACE2 and TMPRSS2 proteins, the presence of high expression levels of both ACE2 and TMPRSS2 in testes highlights the possible vulnerability of men to the virus. Other RNA viruses frequently induce orchitis and result in male infertility. This review evaluates the decline in male fertility and a total of 48 original articles were included for the analysis. We investigated the effects of COVID-19 on male reproductive health and male fertility. There is a strong association between the high number of ACE2 receptors in the testes and the COVID-19 viral loads. SARS-CoV-2 infection negatively affects the male reproductive tract. Human biological tissues, including body fluids and excretions, tissues, and organs showed positive results tests for SARS-CoV-2. A disruption in the balance of male reproductive system hormones is also observed. Male gonads may be potentially vulnerable to SARS-CoV-2 infection, suggesting caution to follow-up and evaluate infected men that have plans to conceive. Further studies are required to determine if this impairment is temporary or permanent, elucidate SARS-CoV-2’s entrance strategies into the testis and how it can affect the semen quality and quantity. We recommend a post-infection follow-up, especially in male patients of reproductive age already having fertility issues.

## SUMMARY

*1. Introduction*

*2. Pathophysiology*

- *2.1 ACE2 as a component of RAAS*

- *2.2 Mechanism of SARS-CoV-2 infection*

- *2.3 ACE2 and TMPRSS2 in the male reproductive system*

- *2.4 Potential pathophysiology of SARS-CoV-2 related male infertility*

*3. Impact of SARS-CoV-2 on male reproductive health: current knowledge*

*4. Conclusion*

## 1. Introduction

Coronavirus disease 2019 (COVID-19) is a viral pneumonia caused by severe acute respiratory syndrome coronavirus 2 (SARS-CoV-2) and it was first identified in Wuhan, China, in December 2019. The virus has spread globally, and the World Health Organization (WHO) declared the outbreak as the first pandemic from a coronavirus on March 11, 2020^1^. As the COVID-19 pandemic emerged, its associated effects on various body systems have come to the surface over the last year. Studies on SARS-CoV-2 have shown that this virus can potentially affect the male population more than the female population. Transmembrane serine protease 2 (TMPRSS2) can enhance angiotensin-converting enzyme 2 (ACE2)-mediated virus entry into human bodies. SARS-CoV-2 seems to have a higher affinity with human ACE2, and for this reason, the complications that may arise from this virus are more severe. TMPRSS2 cleaves ACE2 receptor, therefore the virus enters the host cell by binding to ACE2 to its surface spike protein^2^, causing complications to testes and other parts of the male reproductive system. Testosterone can increase the expression of ACE2 and TMPRSS2, and this could be a possible explanation for the male-dominated infection and higher mortality^3-6^. In this context, impaired spermatogenesis via COVID-19 will decrease the level of testosterone by disturbing cytokines levels, such as TNF-α, IL-4, IL-6, and IL-12, causing further impairment of the sperm count. In addition, IL-4 plays an important role in the activation of the Janus Kinase/Signal Transducer and Activator of Transcription (JAK-STAT) pathway. The deregulation of the IL-4 level alters this pathway and causes male infertility upon COVID-19 infection. Previous studies have shown that inflammatory cytokines play a significant role in spermatogenesis regulation. An imbalance in inflammatory cytokines levels may contribute to infertility in men^7,8^. Given the evidence of SARS-CoV-2 posing a serious threat to the male reproductive system, as well as the risk of causing orchitis, it is of great importance to study COVID-19 possible complications related to male infertility in order to have a better understanding of the correlation of the two^7^. According to the WHO, 48 million couples and 186 million individuals lived with infertility globally as of March 2020. Abnormal sperm function and quality are the leading causes of male infertility^1^. Therefore, this article aims to outline the pathophysiology, the correlation of COVID-19 and male reproductive system and, potential clinical manifestations of the COVID-19 infection with an emphasis on male infertility.

## 2. Pathophysiology

### 2.1. ACE2 as a component of RAAS

**Figure 1:**
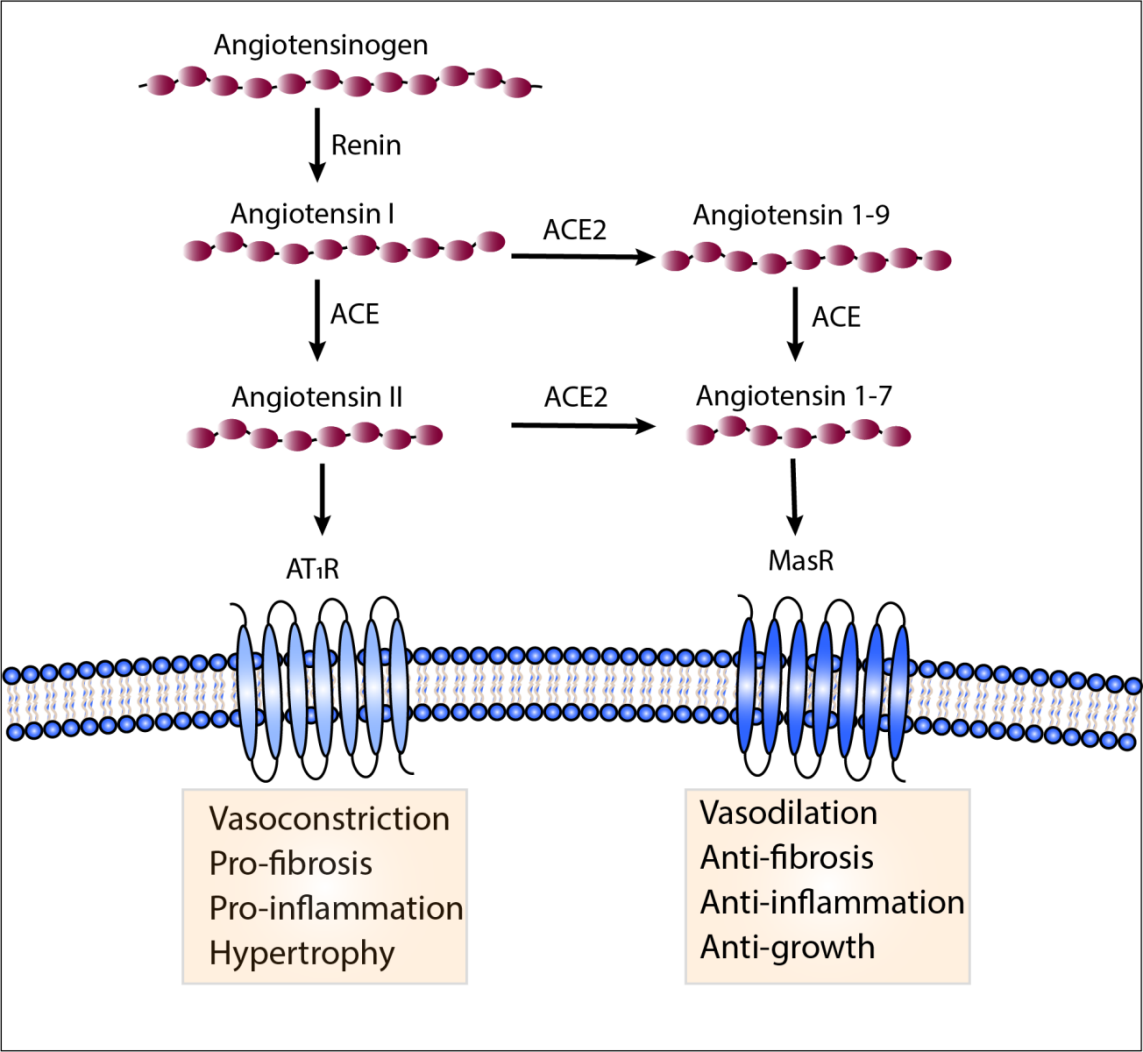
Schematic diagram of the renin-angiotensin-aldosterone system ACE: Angiotensin converting enzyme, ACE2: Angiotensin converting enzyme homologue, AT1R: Angiotensin II Receptor-1, MasR: Mas Receptor.

The Renin-Angiotensin-Aldosterone System (RAAS) is a hormonal cascade that regulates arterial pressure and extracellular volume for homeostatic control of the body. RAAS is initiated by the activation of renin, which converts angiotensinogen to angiotensin I (Ang I). The rest of the pathway is mainly generated by the actions of angiotensin-converting enzyme (ACE) and ACE2 to mediate multiple functions. ACE is a membrane-bound exopeptidase, which removes the C-terminal dipeptide of Ang I to form angiotensin II (Ang II)^9^. ACE2 (also known as ACE-related carboxypeptidase, ACEH) is an ACE homolog. ACE2 is a type-I transmembrane protein that consists of 80S amino acids, encoded by a gene located on chromosome Xp22. ACE2, similarly to ACE, contains two domains: extracellular catalytic domain and intracellular tail. ACE2’s catalytic domain shows 42% similarity to ACE^10,11^. However, unlike ACE, ACE2 does not convert Ang I to Ang II, and its activity is not inhibited by ACE inhibitors (ACEIs)^12^. While ACE catalyzes the cleavage of Ang I to Ang II, ACE2 converts Ang I to angiotensin 1-9 (Ang1-9). Additionally, ACE2 forms angiotensin 1-7 (Ang1-7), a heptapeptide metabolite, from Ang II. Ang II and Ang1-7, the final products of the pathway, act via angiotensin type I receptor (AT1R) and Mas receptor (MasR), respectively. Thus, ACE/Ang II/AT1R and ACE2/Ang1-7/MasR axes demonstrate their functions, which are antagonistic to each other (Figure 1)^13^.

### 2.2 Mechanism of SARS-CoV-2 infection

The SARS-CoV-2 virus enters human cells by using an ACE2 receptor on the host cell membrane. The first step in virus infectivity is the priming of spike (S) glycoprotein, which is a surface enzyme of SARS-CoV-2 virus^2,9^. Spike protein contains two different domains. While the S1 domain directly binds to ACE2 on the host cell membrane, the virus fuses by using its S2 domain^2,14^. However, the expression of ACE2 on the surface cells is not sufficient for the entrance of the virus by itself, and TMPRSS2 also has a notable role in virus infectivity. The priming process of the spike protein is mediated by TMPRSS2. TMPRSS2 cleaves ACE2 receptor, and the virus enters the host cell by binding to ACE2 to its surface spike protein^8^. In the TMPRSS subfamily, TMPRSS4, 11A, 11D, 11D, and 13 also activate S protein other than TMPRSS2 protein to enhance replication of SARS-CoV-2^15,16^.

### 2.3 ACE2 and TMPRSS2 in the male reproductive system

ACE2 has been identified in various human tissues, including, but not limited to the pulmonary, renal, urinary, skin, lymphoid tissue, and hepatobiliary structures^11,17,18^. In male reproductive system, the highest expression of ACE2 was detected in the testes^17^. ACE2 is expressed in spermatogonia, Leydig and Sertoli cells predominantly, whereas spermatids and spermatocytes showed very low expression levels of ACE2^19^.

RAAS components seem to play an important role in male reproduction. ACE2 converts Ang II to Ang1-7 in Leydig cells, therefore it regulates testosterone production and steroidogenesis^20^.

Secondly, ACE2 has been shown to regulate testicular microvasculature to balance interstitial fluid volume^21^.The expression level of ACE2 in testes is age-related, younger males have the highest expression level^22^. ACE2 expression in humans starts with puberty and reaches its maximum level to sustain fertility in men^23^. A study showed that ACE2/Ang1-7/MasR expression level is higher in fertile men when compared to infertile men^24^.

TMPRSS2 has many regulatory roles in cells, such as epithelial sodium homeostasis, angiogenesis and tubulogenesis in microvesicular endothelial cells^25,26^. Being similar to ACE2, TMPRSS2 expression has been shown in all cell clusters in testis, including spermatogonia and spermatids^19^. Most importantly, TMPRSS2 is significantly expressed in the prostate, and was found in prostasomes^27^. Prostasomes are vesicles secreted by epithelial cells of the prostate gland, which are important in sperm motility, protection of the spermatozoa from the acidic vaginal environment, and sperm capacitation. It was suggested that the presence of TMPRSS2 in prostasomes may be essential in the production of healthy sperm^27^.

### 2.4 Potential pathophysiology of SARS-CoV-2 related male infertility

As SARS-CoV-2 virus cell entry mainly depends on ACE2 and TMPRSS2, the presence of high expression levels of both ACE2 and TMPRSS2 in testes highlights the possible vulnerability of male genital tracts to the virus.

**Figure 2:**
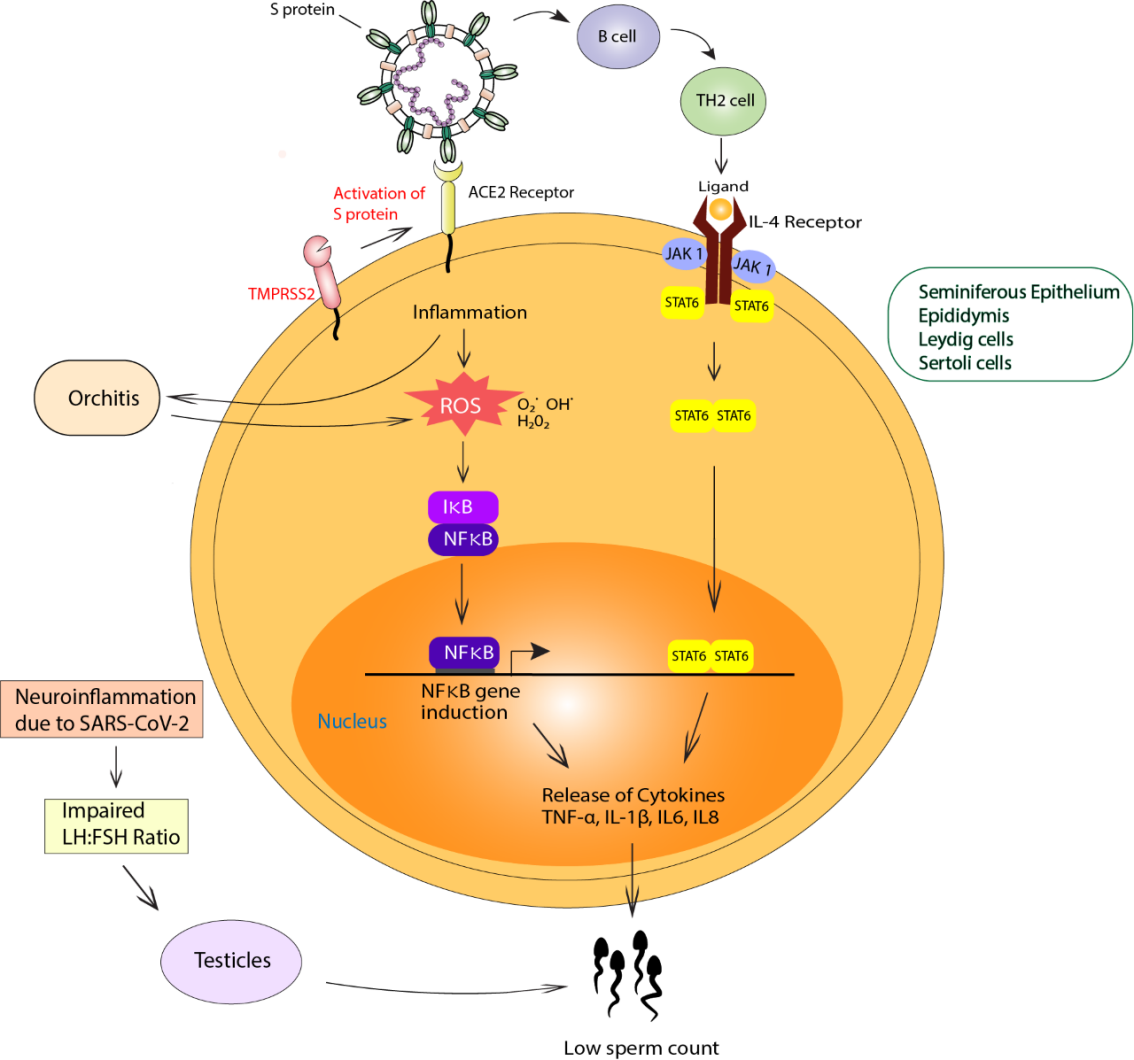
Schematic diagram of the renin-angiotensin-aldosterone system ACE: Angiotensin converting enzyme, ACE2: Angiotensin converting enzyme homologue, AT1R: Angiotensin II Receptor-1, MasR: Mas Receptor.

Here, we are discussing several hypothesized underlying mechanisms of male infertility due to SARS-CoV-2 (Figure 2).

#### A. Direct effect of the virus on reproductive structures

It was suggested that testicular injury may occur directly, induced by the virus, in the setting of COVID-19. In a postmortem examination of the testes from 12 deceased COVID-19 patients, swollen Sertoli cells, vacuolation, and cytoplasmic rarefaction, detachment from tubular basement membranes were observed. Moreover, pathological findings showed significant seminiferous tubular injury, reduced Leydig cells, and mild lymphocytic inflammation^28^.

Previous studies on severe acute respiratory syndrome coronavirus (SARS-CoV) demonstrated that orchitis was associated with the SARS infection. Xu, J. et al. identified pathological changes of testes from six patients who died of SARS. All six cases had orchitis, suggesting that SARS can cause male infertility^29^. As genomic sequences of SARS-CoV and SARS-CoV-2 have 78% homology and share similar mechanisms of infection, this raises the possibility that SARS-CoV-2 may also lead to injury to the testicular cells^30^.

#### B. Causing infertility via impact on the brain and hormonal changes

SARS‐CoV‐2 has been detected in the brain, and it was suggested that the virus has the ability to enter brain cells, since the expression of ACE2 was observed in glial cells and neurons, making them a possible target for SARS‐CoV‐2 infection. The hypothalamic‐pituitary‐gonadal axis (HPG) regulates reproduction by the pulsatile release of gonadotropin-releasing hormone (GnRH) from the hypothalamus. When GnRH is affected by the damage of the central nervous system, it also impacts the release of the follicle‐stimulating hormone (FSH) and the pituitary gland luteinizing hormone (LH), and therefore the sperm production. It is hypothesized that SARS‐CoV‐2 may result in fertility indirectly by its effect on the central nervous system^31^. A recent report from 81 reproductive-aged men with SARS-CoV-2 infection showed that serum LH level was considerably increased, while the ratio of testosterone to LH and the ratio of FSH : LH were significantly decreased in patients with SARS-CoV-2 infection^32^.

#### C. The inflammatory response by cytokines

It was hypothesized that inflammatory response by SARS-CoV-2 may affect fertility in men once SARS-CoV-2 activates the T-helper type 1 (Th1) cells and T-helper type 2 (Th2) cells. Subsequently, Th1 cells produce IFN-γ and promote cellular immunity. On the other hand, Th2 cells express IL-4, which activates the JAK-STAT6 pathway, promotes humoral immunity and apoptosis. It was reported that SARS-CoV-2 increases the level of Th2 cells. Given this evidence, Th2 cells may activate IL-4 release, which causes increased inflammation via the JAK-STAT6 pathway. In turn, this triggers apoptosis and might result in male infertility^7^. A recent study has shown that patients with COVID-19 had low testosterone levels with elevated levels of inflammatory cytokines, such as IL-2 and IFN-γ^33^.

#### D. Oxidative stress-related male infertility

It is well documented that oxidative stress may affect sperm maturation in male reproductive system by causing lipid peroxidation, sperm DNA fragmentation, and apoptosis^34^. Previously, it has been shown that SARS infection might produce reactive oxygen species (ROS), which triggers the NF-kB (nuclear factor kappa-light-chain-enhancer of activated B-cells)-toll-like receptor (mainly TLR-4) pathways. Thus, it results in excessive production of cytokines causing the inflammatory responses, consequently infertility^35^.

## 3. Impact of SARS-CoV-2 on male reproductive health

Recently, several studies are supporting the idea that COVID-19 may negatively affect sperm quality and can reduce fertility in men. This seems to be dependent on the severity of the disease^36^.

As the first coronavirus outbreak was reported in Wuhan, China in December 2019, the first alerts came from the researchers regarding possible fertility issues due to COVID-19. A higher positive rate of ACE2 is reported in the testes of men that have infertility issues. Taking into consideration the physiopathology of SARS-CoV-2, this may indicate that this virus could cause infertility through a pathway activated by ACE2. Qiaoyan Shen at al. studied 9492 testicular single cells that came from normal young males with obstructive azoospermia (OA), and nonobstructive azoospermia males. They found out that men with prior fertility problems may easily be infected by SARS-CoV-2. The expression level of ACE2 was related to age, and the mid-aged had a higher positive rate than young men's testicular cells. Because *ACE2* is highly found in Sertoli cells and Leydig cells, these cells could serve as possible loci of infection and sexual transmission was considered as one of the potential routes of viral transmission^28^. Dr. Ramasamy et al. found and reported impaired sperm function in three of the six testis samples from autopsies of six men who died of COVID-19. They also found evidence of SARS-CoV-2 in the samples of one of the testicles tested, by using electron microscopy. Their findings suggest that men of all ages with COVID-19 symptoms experienced testicular pain. It could be a sign that SARS-CoV-2 has entered the testes. If infected men have fertility plans and/or low testosterone either at present or in the future, they should get evaluated and followed up. Testosterone levels in the blood test should be measured and semen analysis should be done.

But what is the amount of the viral load that needs to be present in the testes in order to be detected in semen? Can SARS-CoV-2 RNA be sexually transmitted^37^? Holtmann et al.’s cohort study, early during this pandemic did not detect SARS-CoV-2 RNA in semen of recovered and acute COVID-19 positive men. Their study suggested viral transmission has not been observed during sexual contact and assisted reproductive techniques. No RNA was detected by means of RT-PCR in the semen, including semen samples from two patients with an acute COVID-19 infection. Subjects with a moderate infection showed an impairment of sperm quality^38^.

However, He et al. reported that human biological tissues, including body fluids and excretions (sputum, sperm, saliva, nasal discharge, umbilical cord blood), tissues, and organs showed positive results tests for COVID-19^39^.

In addition, Achua et al. studied the COVID-19 positive autopsy patients ages ranging from 20 to 87 years old (n=6, mean=56 years old). In one patient’s autopsy, they found out macrophage and lymphocyte infiltration on H&E histomorphological analysis. In COVID-19 positive patients with normal spermatogenesis, ACE2 receptor expression was noted to be significantly decreased when compared to COVID-19 positive patients with impaired spermatogenesis (p<0.05). The presence of the virus can still be identified in the testis after patients have seroconverted^40^.

According to a very recent perspective, longitudinal cohort study of 84 men with laboratory-confirmed COVID-19 and 105 men without the disease in Iran, there were observed changes in ACE2 activity, apoptotic variables, semen quality, markers of inflammation and oxidative stress. All were evaluated at 10-day intervals for up to 60 days. Among 84 patients who tested positive for COVID-19, 1.2% (1/84) were diagnosed as ‘mild type’, 27.4% (23/84) as ‘moderate type’, 32.1% (27/84) as ‘severe type’, and 39.3% (33/84) as ‘critical type’. Corticosteroids and antiviral therapies used were 44.0% (37/84) and 69.0% (58/84), respectively.

The disease severity accompanied perturbations in the seminal cytokines. The magnitude of the perturbations in the ACE2 activity was related to the disease severity. This is another study reporting that the disease severity is associated with changes in semen quality parameters. A significant negative correlation was observed between semen quality parameters with ACE2 activity, pro- and anti-inflammatory cytokines, apoptotic variables, and ROS in both groups. This study demonstrated that COVID-19 infection causes significant impairments of male reproductive function. In detail, compared to the age-matched healthy controls, male patients recovering from COVID-19 had significantly higher levels of seminal ACE2 enzymatic activity, pro- and anti-inflammatory cytokines, and apoptotic variables, as well as lower levels of superoxide dismutase (SOD) activity at the baseline. This report provides another evidence to date that COVID-19 infection impairs semen quality and male reproductive potential. It reports that these perturbations persisted over time and were associated with significant impairments in semen quality parameters. They suggest that the semen of the patients should be considered as a vulnerable route to COVID-19 infection. Because of that, male patients recovering from COVID-19 should be evaluated and followed up. It is possible that they may develop a transient state of male subfertility, like those with oligoasthenoteratozoospermia^41^.

The presence of the ACE2 receptor on germ cells, Leydig cells, and Sertoli cells recently indicated the testis as a potential target of SARS-CoV-2 infection. The serine protease receptor TMPRSS2 is also present in male reproductive tissues and plays an important role in helping the entry of SARS-CoV-2. Evidence indicates a possible long-term effect of the pathogenicity of SARS-CoV-2 infection on testicular tissue, which may impact reproductive performance. These studies support the idea that the possibility of sexual transmission of SARS-CoV-2 cannot be ruled out^42-44^. In addition, one of the major symptoms of the COVID-19 pandemic is a very high fever with a sudden surge in body temperature. Fever can negatively affect male fertility. Testicular heat stress determines an increase of reactive oxygen species, causing oxidative stress and sperm DNA fragmentation^45,46^.

In some patients, viral infiltration into the semen may also be manifested by increased viremia. There is also a possibility for hyperthermia due to fever, hypoxia, secondary infection, and steroids being the key mediators of testicular damage in SARS-CoV-2 patients^28,47^. In addition, a novel case of SARS-CoV2 bilateral orchitis is reported in a previously healthy 37-year-old male who presented for testicular pain with constitutional symptoms. This could be another mechanism of how SARS-CoV2 can affect spermatocyte function^48^.

Ma et al.’s retrospective study compared the sex-related hormones between 81 reproductive-aged men with SARS-CoV-2 infection and 100 age-matched healthy men. The study showed that serum luteinizing hormone (LH) was significantly increased, but the ratio of testosterone to LH and the ratio of follicle-stimulating hormone (FSH) to LH were dramatically decreased in males infected with COVID-19^32^. Furthermore, they also reported that compared to the control group, COVID‐19 patients had significantly higher serum LH (p < .0001). They reported there is no statistical difference in serum FSH (p = .5585), serum testosterone (p = .1886) or between the two groups. However, the ratios of testosterone : LH (p < .0001) and FSH : LH (p < .0001) were decreased in the group of the individuals with COVID‐19. No significant difference was observed in estrogen (p = .9364) or testosterone: estrogenE ratio (p = .7096)^32^.

Different viruses, such as mumps virus, HIV in humans, and Zika virus in mice, frequently induce orchitis and result in male infertility. The mechanisms they use to cause damage in male infertility are not well understood. In addition, other viruses, including influenza, coxsackie, dengue, rubella and echovirus viruses, may also cause orchitis and impair testicular functions. All of these viruses belong to RNA viruses^49^ and SARS-CoV-2 being a positive-stranded RNA (+ssRNA) virus might be causing orchitis through the same pathways^50^.

All these reports suggest that SARS-CoV-2 may target the testis, and potentially induce testicular damage and affecting testis function, in part through secondary immunological and inflammatory responses. Furthermore, these damage and functional alterations may lead to infertility^37^.

## 4. Conclusion

In conclusion, the reported cases in the literature suggest that SARS-CoV-2 infection negatively affects the male genital system in direct or indirect ways. Even though mechanisms of SARS-CoV-2-mediated male infertility are not clear, possible pathologies were hypothesized and discussed by researchers in several studies to reveal how this virus impairs semen and can cause male infertility. The impairment of the semen is frequently reported since the beginning of the pandemic more than 1 year ago. Further studies are required to determine if this impairment is temporary or permanent, elucidate the ways SARS-CoV-2 entrance into the testis and how it damages the semen. It is not clear yet what is the viral load needed for SARS-CoV-2 to be transmitted through the semen of infected men. These studies suggest that infection with SARS−CoV-2 may affect male fertility. We recommend that health care providers should recognize COVID-19 in males and do a post-infection follow-up, especially in male patients of reproductive age already having fertility issues.
